# Value of circulating cell-free DNA analysis as a diagnostic tool for breast cancer: a meta-analysis

**DOI:** 10.18632/oncotarget.15775

**Published:** 2017-02-28

**Authors:** Ziqiang Lin, James Neiswender, Bin Fang, Xuelei Ma, Jing Zhang, Xiuying Hu

**Affiliations:** ^1^ State Key Laboratory of Biotherapy and Cancer Center, West China Hospital, West China Medical School, Sichuan University, Chengdu, Sichuan 610041, China; ^2^ Program in Molecular Medicine, Department of Molecular Cell and Cancer Biology, University of Massachusetts Medical School, Worcester, Massachusetts 01655, USA; ^3^ Sixth Unit of Internal Medicine, Dujiangyan Medical Center, Dujiangyan, Sichuan 611830, China; ^4^ Department of Nursing, West China Hospital, Sichuan University, Chengdu, Sichuan 610041, China

**Keywords:** cell-free DNA, breast cancer, diagnosis, meta-analysis, accuracy

## Abstract

**Objectives:**

The aim of this study was to systematically evaluate the diagnostic value of cell free DNA (cfDNA) for breast cancer.

**Results:**

Among 308 candidate articles, 25 with relevant diagnostic screening qualified for final analysis. The mean sensitivity, specificity and area under the curve (AUC) of SROC plots for 24 studies that distinguished breast cancer patients from healthy controls were 0.70, 0.87, and 0.9314, yielding a DOR of 32.31. When analyzed in subgroups, the 14 quantitative studies produced sensitivity, specificity, AUC, and a DOR of 0.78, 0.83, 0.9116, and 24.40. The 10 qualitative studies produced 0.50, 0.98, 0.9919, and 68.45. For 8 studies that distinguished malignant breast cancer from benign diseases, the specificity, sensitivity, AUC and DOR were 0.75, 0.79, 0.8213, and 9.49. No covariate factors had a significant correlation with relative DOR. Deek's funnel plots indicated an absence of publication bias.

**Materials and Methods:**

Databases were searched for studies involving the use of cfDNA to diagnose breast cancer. The studies were analyzed to determine sensitivity, specificity, positive likelihood ratio, negative likelihood ratio, diagnostic odds ratio (DOR), and the summary receiver operating characteristic (SROC). Covariates were evaluated for effect on relative DOR. Deek's Funnel plots were generated to measure publication bias.

**Conclusions:**

Our analysis suggests a promising diagnostic potential of using cfDNA for breast cancer screening, but this diagnostic method is not yet independently sufficient. Further work refining qualitative cfDNA assays will improve the correct diagnosis of breast cancers.

## INTRODUCTION

Breast cancer is the most frequently diagnosed cancer worldwide and the leading cause of cancer death among females, accounting for 23% of total cancer cases and 14% of cancer deaths [1]. Despite increasing incidence, mortality from breast cancer has declined over the past decade [2]. A considerable proportion of the decrease in mortality is attributed to early diagnostic methods, such as modern digital mammography. However, 13% of breast cancers are undetectable by mammography affected by tumor size and age of patients [3, 4]. Currently used biomarkers with unsatisfactory accuracy, such as cancer antigen CA15-3 and carcinoembryonic antigen (CEA), have been recommended against for accurately diagnosing breast cancer [5, 6]. Therefore, development of new technologies with enhanced sensitivity and specificity to detect and diagnose breast cancer is in critical demand.

Circulating cell-free DNA (cfDNA) is fragmented DNA originating from cancer cells through the processes of necrosis and apoptosis [7]. The cfDNA containing specific mutations, copy number alterations, and structural variants prevail in a wide range of cancers, including pancreatic, ovarian, colorectal, bladder, breast cancers, and other pathologies with rapid cell turnover [8–11]. Through highly sensitive techniques to detect abnormalities of circulating cfDNA, including digital PCR-based [8, 12, 13] and massive sequencing-based technologies [8, 9, 14], it is now feasible to improve early screening and surveillance of breast cancer.

The detection of alternations in circulating cfDNA present in breast cancer patients has led to a wealth of studies that have analyzed the genetic and epigenetic character of these alterations, including microsatellite instability and aberrant DNA methylation in plasma or serum [15]. Many studies have addressed the potential value of circulating cfDNA assays as a repeatable and non-invasive “liquid biopsy” for breast cancer [7, 16]. However, these results of such studies have varied, but have not been systematically reviewed. Hence, the aim of this meta-analysis was to quantitatively evaluate the diagnostic efficiency of circulating cfDNA assays for breast cancer screening.

## RESULTS

### Database analyses

**Figure 1:**
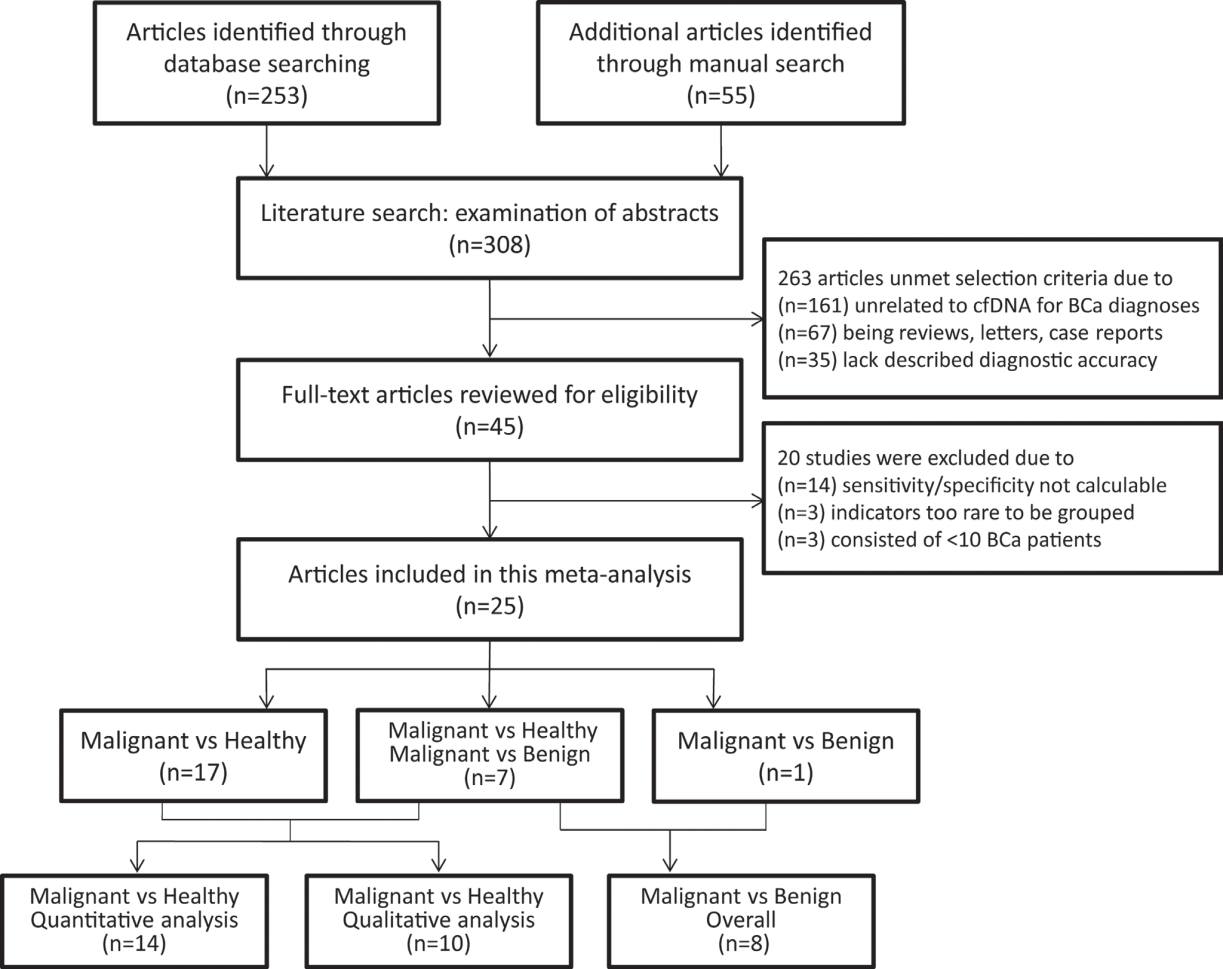
Study identification, inclusion, and exclusion for meta-analysis BCa = breast cancer; cfDNA = cell-free DNA; vs = versus.

In primary review, a total of 45 publications dealing with abnormal concentration [8, 17–36], methylation alterations [37–44], microsatellite instability [45–50] and other characteristics [10, 14, 51–58] of plasma or serum DNA for the diagnosis of breast cancer were retrieved. After full-text review, 20 studies [8, 10, 14, 29–35, 42–44, 48–50, 56–59] were excluded because they did not allow the calculation of sensitivity or specificity, included very rare indicators, or consisted of less than 10 breast cancer patients (Figure 1).

In total, 25 eligible studies [17–28, 36, 38–41, 45–47, 51–55, 60] were included in the analyses (Table 1), comprising 1705 histologically diagnosed breast cancer patients, 1079 healthy controls, and 234 patients with benign breast diseases. A majority of 1959 subjects were from the United States and European countries, with the remaining 979 participants from Asian areas (China, Thailand and Israel) and 80 from Africa (Egypt). Of 25 studies, 15 assessing abnormal concentrations of circulating cfDNA were classified as the quantitative analysis group, while 10 trials evaluating multi-gene methylation alterations, allelic imbalances and genome-wide aberrations represented the group of qualitative analysis. In addition to assessments of 24 studies using health individuals as control [17–28, 38–41, 45–47, 51–55], a diagnostic assessment was also conducted in 8 studies that included benign breast diseases as controls, half of which are quantitative studies while the remain are qualitative [18, 21–23, 36, 39, 40, 45].

**Table 1:** Summary of included studies

Study/year	Country	No.of BCa/BD/Ctrl	Sample	Assay methods	Cutoff of BCa vs Ctrl (BCa vs BD)	Sens/Spec of BCa vs Ctrl (%)	Sens/Spec of BCa vs BD (%)	Groups
Leon/1977	USA	32/-/55	serum	RIA	50 ng/ml	38/93	-	quantitative
Chen/1999	Switzerland	21/2/10	serum	MA	LOH reduced by 30%	48/100	48/50	qualitative
Shaw/2000	UK	71/-/9	plasma	MA	LOH	31/100	-	qualitative
Silva/2002	Spain	142/-/35	plasma	MA	LOH reduced by 75%	42/100	-	qualitative
Muller/2003	Austria	86/-/10	serum	MSP	hypermethylation	22/90	-	qualitative
Dulaimi/2004	USA	34/8/20	serum	MSP	hypermethylation	76/100	76/100	qualitative
Gal/2004	UK	96/-/24	serum	RT-qPCR	100 ng/ml	72/88	-	quantitative
Skvortsova/2006	Russia	20/15/10	plasma	MSP	hypermethylation	95/100	95/40	qualitative
Huang/2006	China	61/33/27	plasma	RT-qPCR	19 ng/ml (22 ng/ml)	95/89	93/67	quantitative
Umetani/2006	USA	51/-/51	serum	RT-qPCR	integrity of 0.17	69/80	-	quantitative
Korshunova/2008	USA	21/-/21	serum	BPS	cytosine-methylation	95/100	-	qualitative
Catarino/2008	Portugal	175/-/80	plasma	RT-qPCR	106 ng/ml	43/91	-	quantitative
Kohler/2009	Switzerland	52/26/70	plasma	RT-qPCR	1866GE/ml (463282GE/ml)	81/69	53/87	quantitative
Beck/2010	USA	10/-/87	serum	NGS	repetitive elements	90/95	-	qualitative
Roth/2011	Germany	63/20/28	serum	ELISA	-	72/86	13/65	quantitative
Gong/2012	China	200/100/100	serum	RT-qPCR	471 ng/ml	95/92	95/90	quantitative
Dawson/2013	UK	30/-/22	plasma	TADS	-	97/100	-	qualitative
Stotzer/2014	Germany	112/-/28	plasma	RT-qPCR	-	94/95	-	quantitative
Madhavan/2014	Germany	82/-/100	plasma	RT-qPCR	-	72/78	-	quantitative
Kirkizlar/2015	USA	11/-/30	plasme	NGS	0.45% AAI	73/100	-	qualitative
Tangvarasittichai/2015	Thailand	100/-/100	plasma	RT-qPCR	100 ng/ml	97/93	-	quantitative
Zhang/2015	China	100/-/104	serum	RT-qPCR	RC of 0.30	80/68	-	quantitative
Wu/2015	USA	47/-/42	plasma	RT-qPCR	T/R ratio of 91.40	92/75	-	quantitative
Agassi/2015	Israel	38/-/16	serum	FSGS	600 ng/ml	72/75	-	quantitative
Mahmoud/2015	Egypt	50/30/-	serum	RT-qPCR	2236 copy/ul	-	76/70	quantitative

BCa = patients with breast cancers; BD = patients with benign breast diseases; Ctrl = healthy controls; TP = true positive; FP = false positive; FN = false negative; TN = true negative; RIA = radioimmunoassay; MA = microsatellite analysis; LOH = loss of heterozygosity; MSP = methylation specific PCR; RT-qPCR = real-time quantitative PCR; RC = relative concentration; T/R = copy number of telomere relative to copy number of LINE reference sequence; ELISA= enzyme linked immunosorbent assay; BPS = bisulphate pyrosequencing; NGS = next-generation sequencing; AAI = average allelic imbalance; TADS = tagged-amplicon deep sequencing; FSGS = fluorochrome SYBR Gold stain.

### Diagnostic accuracy

**Figure 2:**
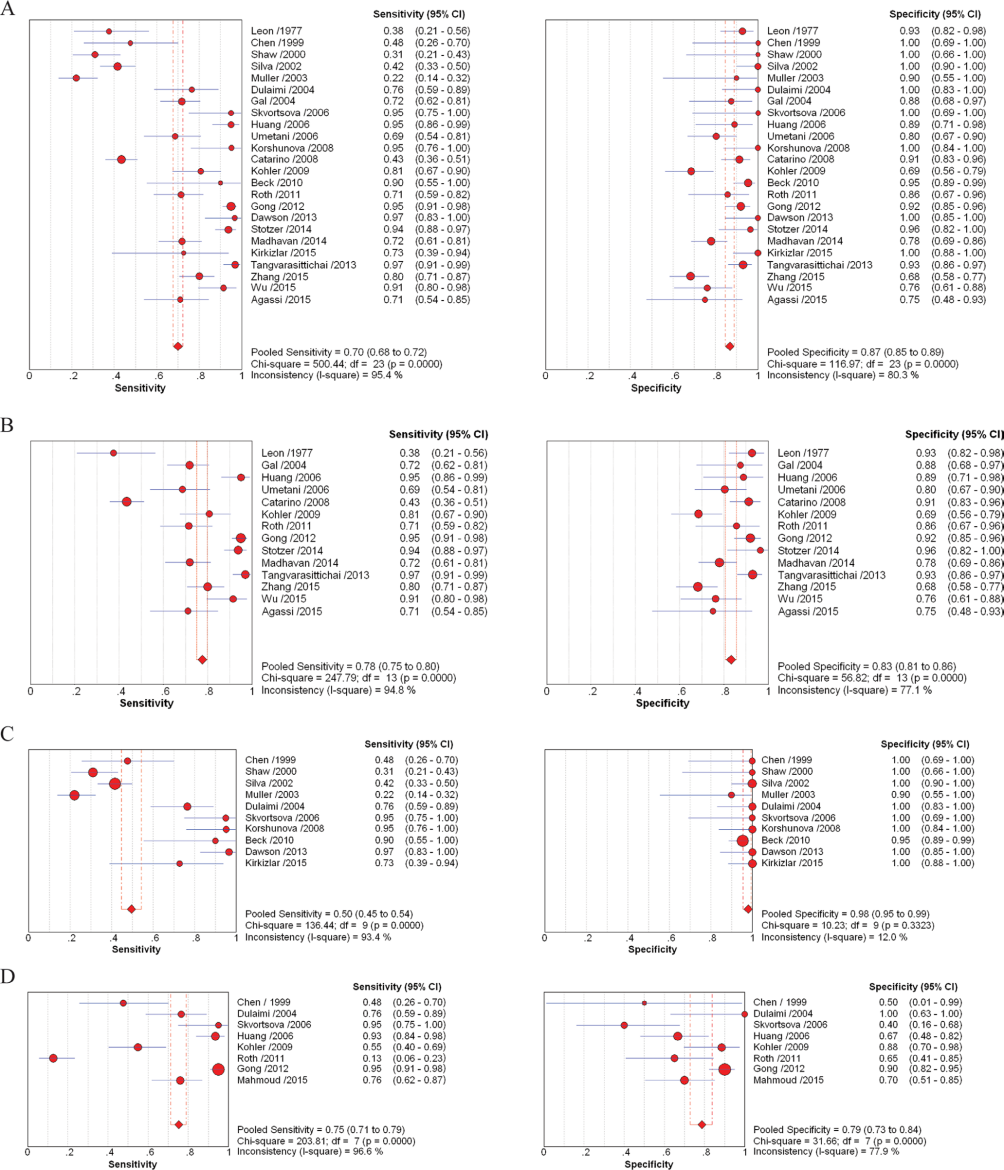
Forest plots of estimates of sensitivity and specificity for different cell-free DNA assay groups Forest plots of sensitivity and specificity for assays of circulating cell-free DNA in the diagnosis between healthy individuals and breast cancer patients (**A**), and between benign breast disease and breast cancer patients (**D**). Forest plots of sensitivity and specificity for methodological groups using quantitative (**B**) and qualitative (**C**) analysis of circulating cell-free DNA in the diagnosis of breast cancer. 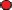 = points estimates of sensitivity and specificity from each study; error bars = 95% CI.

Sensitivity and specificity, PLR, NLR, and DOR are indicators applied to estimate diagnostic accuracy. Generated by integrating 24 trials, the overall sensitivity and specificity of cfDNA assays, to distinguish breast cancer patients and healthy individuals, were 0.70 (95% CI, 0.68–0.72) and 0.87 (95% CI, 0.85–0.89), respectively. PLR was 6.22 (95% CI, 4.31–8.99), NLR was 0.25 (95% CI, 0.17–0.36), and DOR was 32.31 (95% CI, 17.35–60.18) (Figure 2A). To determine how methodology affected diagnostic accuracy, we further analyzed two groups that employed quantitative (testing cfDNA concentrations) and qualitative (evaluating multi-gene methylation, allelic imbalances, and genome-wide aberrations) methodologies.

The estimates of sensitivity and specificity of the 14 quantitative analyses of cfDNA for breast cancer diagnosis (Figure 2B) were 0.78 (95% CI, 0.75–0.80) and 0.83 (95% CI, 0.81–0.86), respectively. The value for PLR was 4.83 (95% CI, 3.37–6.91), and NLR was 0.22 (95% CI, 0.13–0.35). The DOR value was 24.40 (95% CI, 12.07–49.31). To further evaluate the diagnostic accuracy of quantitative PCR-based assays, three quantitative studies using radioimmunoassay [54], ELISA [22] and fluorochrome SYBR Gold stain [55] were excluded for further analysis. The value for sensitivity, PLR and DOR increased to 0.79 (95% CI, 0.77–0.82), 5.07 (95% CI, 3.32–7.75) and 31.91 (95% CI, 13.65–74.62); while specificity and NLR declined to 0.83 (95% CI, 0.80–0.85) and 0.17 (95% CI, 0.09–0.32) (Supplementary Table 1).

Figure 2C shows the sensitivity and specificity generated from 10 qualitative analyses, including methylation PCR, microsatellite analysis and sequencing, in diagnosis of breast cancer. The sensitivity and specificity were 0.50 (95% CI, 0.45–0.54) and 0.98 (95% CI, 0.96–0.99); PLR was 16.52 (95% CI, 8.65–31.58), NLR was 0.32 (95% CI, 0.19–0.54), and DOR was 68.45 (95% CI, 19.29–242.85). When we excluded four studies with comparatively low sensitivity [38, 45–47], the sensitivity increased to 0.88 (95% CI, 0.81–0.93), while specificity dropped slightly to 0.98 (95% CI, 0.95–0.99); PLR was 24.46 (95% CI, 11.38–52.58), NLR was 0.14 (95% CI, 0.07–0.28), and DOR was 256.60 (95% CI, 77.40–850.69) (Supplementary Table 1). In addition, the diagnostic accuracy of cfDNA assays between benign diseases and malignant tumors was estimated. Sensitivity and specificity were 0.75 (95% CI, 0.71–0.79) and 0.79 (95% CI, 0.73–0.84), PLR was 2.40 (95% CI, 1.13–5.12), NLR was 0.29 (95% CI, 0.12–0.74), and DOR was 9.49 (95% CI, 1.76–51.03) (Figure 2D).

**Figure 3:**
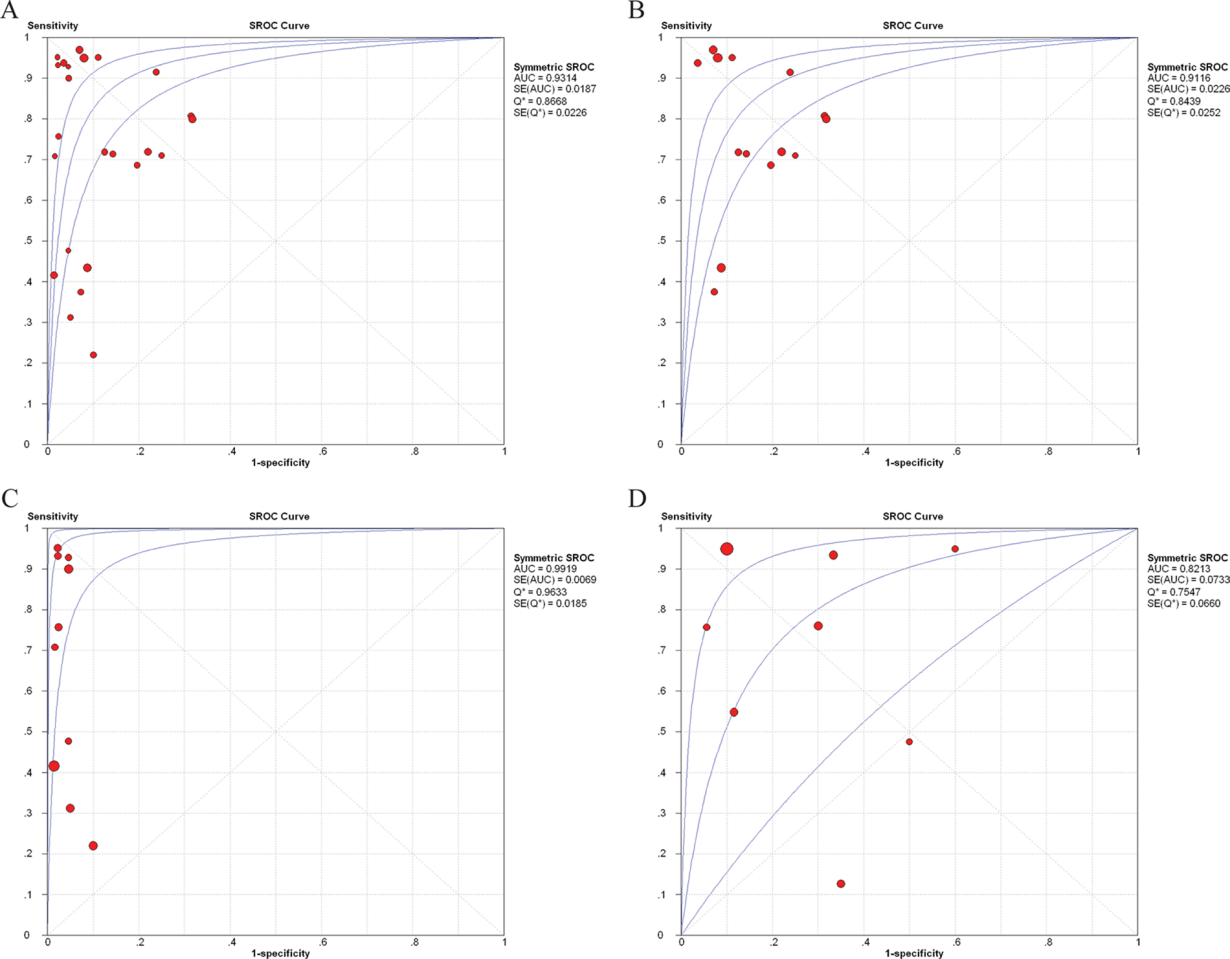
SROC curves for cell-free DNA assays in diagnosis of breast cancer SROC curves for cell-free DNA assays in diagnosis of breast cancer. SROC curves for cell-free DNA assays in diagnosis between healthy individuals and breast cancer patients (**A**), and between benign breast disease and breast cancer patients (**D**). SROC curves for methodological groups using quantitative (**B**) and qualitative (**C**) analysis of cell-free DNA in the diagnosis of breast cancer. 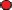 = each study in the meta-analysis (the size of each study is indicated by the size of the solid circle); red line = weighted regression; and blue line = unweighted regression. SROC curves summarize the overall diagnostic accuracy. The confidence ellipse indicates that the mean values for sensitivity and specificity were more likely to be in this region. The prediction ellipse (increased uncertainty) indicates that individual values for sensitivity and specificity were more likely to be in this region.

Furthermore, the observed data, together with the confidence and predictive ellipses, are presented in SROC curves to determine their diagnostic heterogeneity. The satisfactory diagnostic performance for cfDNA assays for diagnosis of cancer patients from healthy individuals was demonstrated by the SROC curve in Figure 3A. The AUC was 0.9314, the LRT_I^2^ statistic was 78.60%, the LRT_Q (χ^2^) was 107.52 (*p* < 0.001), and the Spearman correlation coefficient was -0.061 (*p* = 0.777), indicating considerable heterogeneity between studies caused by non-threshold effects.

SROC curves were also applied for the methodological groups. In the quantitative group (Figure 3B), the AUC was 0.9116 (0.9193 specific for qPCR assays), indicating acceptable levels of diagnostic accuracy. The LRT_I^2^ value was 84.30%, presenting some evident heterogeneity in these studies. The LRT_Q (χ^2^) was 82.76 (*p* < 0.001) and Spearman correlation coefficient was −0.191 (*p* = 0.513), indicating that the heterogeneity was likely the result of non-threshold effects. In the qualitative group, the AUC was 0.9919 (0.9886 when four aforementioned studies were excluded; seen in Figure 3C), suggesting higher diagnostic accuracy compared with the quantitative group. The LRT_ I^2^ was 49.80% and the LRT_Q (χ^2^) was 17.94 (*p* = 0.036), and Spearman correlation coefficient was -0.383 (*p* = 0.275), revealing no significant heterogeneity.

The SROC curve of cfDNA assays for diagnosis of cancer patients from benign disease populations generated an AUC of 0.8213, the LRT_I^2^ was 91.20%, the LRT_Q (χ^2^) was 79.25 (*p* < 0.001), and the Spearman correlation coefficient was −0.096 (*p* = 0.821), indicating heterogeneity between studies was caused by non-threshold effects (Figure 3D).

### Meta-regression analysis and publication bias

To reveal sources of heterogeneity resulting from non-threshold effects, we assessed major characteristics of these studies, including “publication year (recent 5 years)”, “country (Asian regions)”, “case number (≥ 100 cases)”, “sampling (plasma)”, and “assay methods (microsatellite/ methylation/ sequencing analysis as qualitative analyses; qPCR/ the rest as quantitative analyses)”. These characteristics were used in the meta-regression analyses to assess their effects on the RDOR in the diagnosis of breast cancer. The results suggested that none of the methodological covariates may produce major heterogeneity (*p* > 0.05) among different groups (Table 2).

**Table 2:** Weighted meta-regression of effects of methodological characteristics on diagnostic accuracy of cfDNA

Covariates	Coefficient	RDOR (95%)	*P* value	Coefficient	RDOR (95% CI)	*P* value
	Breast cancer versus healthy controls: overall analysis			Breast cancer versus benign disease: overall analysis		
Country	0.748	2.11 (0.42–10.59)	0.346	2.846	17.21 (0.63–472.03)	0.0802
Year	0.705	2.02 (0.49–8.40)	0.3152	−0.472	0.62 (0.01–36.47)	0.7862
Case No.	0.859	2.36 (0.50–11.14)	0.2633	3.383	29.47 (0.34–2537.67)	0.1125
Sampling	−0.835	0.43 (0.11–1.79)	0.2346	−0.758	0.47 (0.01–27.94)	0.6660
Method	−1.003	0.37 (0.08–1.74)	0.1946	−1.641	0.19 (0.03–1.32)	0.0811
	**Breast cancer versus healthy controls: quantitative analysis**			**Breast cancer versus healthy controls: qualitative analysis**		
Country	1.313	3.72 (0.70–19.88)	0.1136	–	–	–
Year	0.968	2.63 (0.47–14.78)	0.2450	2.007	7.44 (0.18–308.17)	0.2491
Case No.	1.414	4.11 (0.75–22.55)	0.0955	−0.349	0.71 (0.00–115.88)	0.8787
Sampling	−0.907	0.40 (0.07–2.34)	0.2831	−0.712	0.49 (0.02–10.14)	0.6024
Method	−0.005	1.00 (0.09–11.36))	0.9965	1.414	4.11 (0.84–20.12)	0.0741

**Figure 4:**
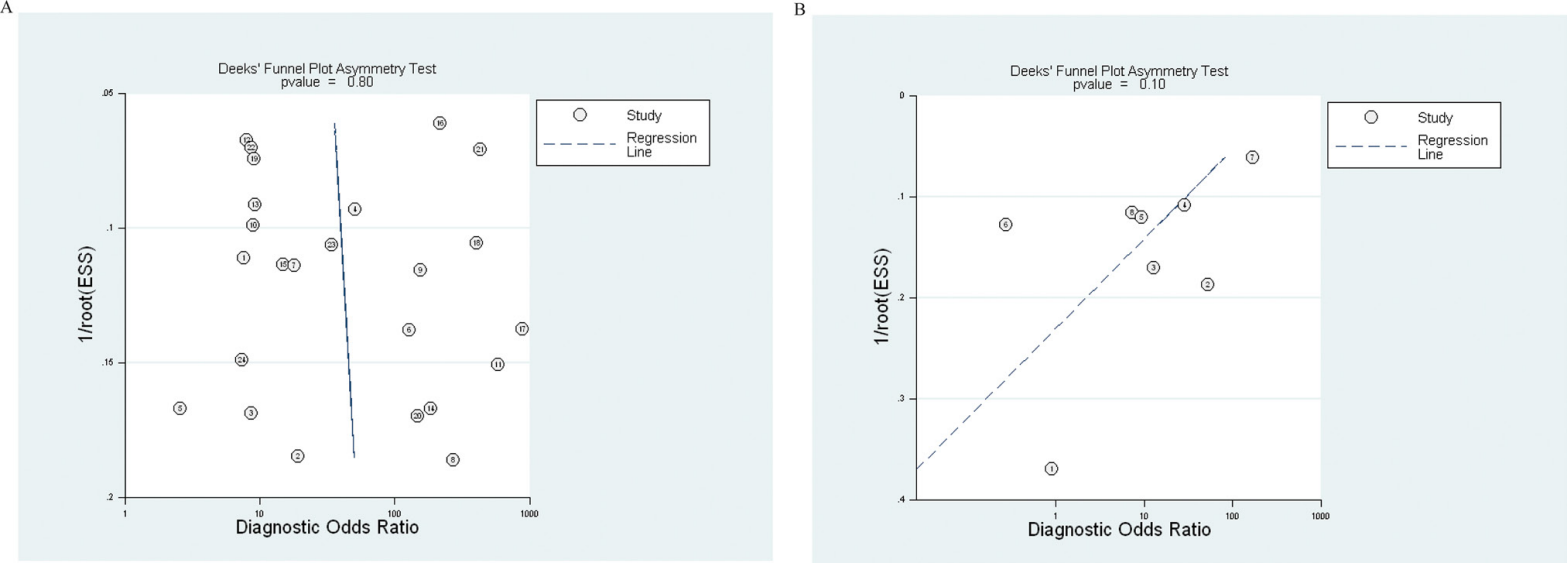
Funnel graphs for the assessment of potential publication bias in cell-free DNA assays Funnel graphs for the assessment of potential publication bias in cell-free DNA assays to distinguish breast cancer patients vs. healthy individuals (**A**), and assays to distinguish breast cancer vs benign breast disease patients (**B**). The funnel graph performs linear regression of log odds ratios on inverse root of effective sample size (ESS). 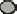 = each study in the meta-analysis; center line = regression line. The results of Deek's funnel plot asymmetry test for publication bias of studies to distinguish breast cancer patients vs healthy individuals (A) and studies to distinguish breast cancer patients vs benign disease patients (B) were not significant (*p* = 0.80 and 0.10, respectively).

Publication biases in these diagnostic analyses were evaluated using the Deek's funnel plot asymmetry test. The DOR of all 24 studies to distinguish breast cancer patients from healthy individuals aligned in a fairly symmetric linear regression with a coefficient of 2.71 (95% CI, −19.22–24.64; *p* = 0.80). A non-zero slope coefficient is suggestive of significant study bias when *p* < 0.10. Thus, publication bias was not significant in these studies. The test result for 8 studies applying cfDNA to distinguish breast cancer from benign breast diseased patients was also presented. The coefficient was −25.98 (−59.20–7.14) and the *p*-value was 0.10. This comparatively less symmetric plot of linear regression also indicated no significant publication biases for this group (Figure 4).

## DISCUSSION

Our work is the first meta-analysis to calculate the overall accuracy of circulating cell-free DNA assays for detection of breast cancer. The sensitivity and specificity of cfDNA assays based on 24 primary studies were 0.70 and 0.87 respectively, indicating that a correct diagnosis could often be made through these assays. The AUC calculated for SROC curves was 0.9314, well above common standards for diagnosis (> 0.8). Among other serum-based breast cancer markers, CA15-3 exhibited acceptable sensitivity, specificity, and AUC at 0.73, 0.85, and 0.78, respectively [61]; the estimated sensitivity, specificity, and AUC of human epidermal growth factor receptor 2 (HER2) were measured at 0.51, 0.86, and 0.65 [62]. The accuracy of the cfDNA assay appears to be modestly stronger than either of these traditional markers, yet few studies have directly compared the diagnostic performance of cfDNA with other biomarkers.

In grouped analyses, the sensitivity, specificity, and AUC of 14 quantitative studies were slightly lower (0.78, 0.83, and 0.9116) than those of the overall group. When only PCR-based studies were included, the sensitivity and AUC subtly increased, suggesting that qPCR achieved marginally improved diagnostic efficiency compared to the other quantitative assays [22, 54, 55]. However, the limited number of other quantitative studies hindered analysis of their diagnostic values. In the group of 10 qualitative studies, the sensitivity dramatically reduced to 0.50; whereas, the specificity and AUC notably improved to 0.98 and 0.9919. The qualitative group included studies applying various methods to detect epigenetic and genetic abnormalities of cell-free DNA. Among these qualitative methods, microsatellite analyses [45–47] and methylation specific PCR [38] produced very limited sensitivity ranging from 0.22–0.48. By removing these, the remaining studies achieved satisfactory specificity as high as 0.88. The more modern high-throughput molecular methods, including next-generation sequencing and multiplexed PCR [51, 53], have proven to be potent strategies for breast cancer screening.

To further evaluate diagnostic effectiveness, we also analyzed the diagnostic odds ratio (DOR), which is a single indicator of test accuracy [63]. The value of DOR > 10 indicates good discriminatory test performance. In this meta-analysis, the DOR for cfDNA assays to discriminate breast cancer cases from healthy controls was 32.31, while the DOR to distinguish malignant breast tumors and benign breast diseases was much lower at 9.49. The fact that cfDNA may elevate among patients with benign diseases limited the potential of the quantitative cfDNA assay as a tool to discriminate benign hyperplasia and malignancy [21, 22]. The DOR of qualitative assays of cfDNA (68.45) is significantly higher than that of quantitative assays (24.40). The DOR was further improved to 256.60 with 4 earlier published low-sensitive studies excluded. In established studies applying cfDNA for cancer diagnoses, the DOR of quantitative analyses in lung [64], ovarian [65], and hepatocellular cancer (HCC) [66] were 20.33, 26.05, and 16.35, respectively; the DOR of qualitative methods for HCC diagnosis was 19.49. The DOR of qualitative cfDNA analysis for breast cancer was notably higher than that of HCC, and the DOR of quantitative analysis was comparable with those of other cancer types, indicating a strong ability to correctly diagnose breast cancer using cfDNA assays, especially qualitative molecular methods.

PLR and NLR were also presented to measure overall diagnostic accuracy [67]. Likelihood ratios of a PLR > 10 and an NLR < 0.1 indicate high accuracy. The group of quantitative assays had a PLR value of 6.22, suggesting that patients with breast cancer have an approximately 6-fold higher chance of being cfDNA assay-positive compared with healthy controls. The NLR of quantitative analyses was found to be 0.25, implying that the probability for cases with negative test results to have breast cancer is one fourth. These data suggest that circulating DNA assay results should not be used alone as a biomarker to make a breast cancer diagnosis. The qualitative analysis was more promising with a PLR of 16.52 and NLR of 0.32, indicating that an approximately sixteen times greater chance of a breast cancer case being indicated by a positive test result, but a 32% error rate would be present when a healthy individual was determined in the negative test. According to the results, cfDNA assessments may be applied in early detection of breast cancer, but as an auxiliary test it should be combined with cytological or histological examination of breast tissue to ensure correct diagnosis.

Considering the effect of publication bias, the results could have been biased if positive results were more likely to be published. The Deek's funnel plot asymmetry test compared diseased and healthy groups, or malignant and benign groups, but did not indicate publication bias. Although we found a statistically significant heterogeneity for sensitivity, specificity, PLR, NLR, and DOR among these studies, we found none of the study characteristics including publication year, country, case number and assay types of these studies to represent a major source of heterogeneity. The heterogeneity could have been derived from differences on other methodological characters, such as prospective/retrospective designs and TNM staging of patients enrolled, which were not included in meta-regression analysis due to incomplete information provided by the primary studies. In addition, two studies included less than 20 cancer patients [51, 53], which may have contributed to the poor robustness. Despite significant heterogeneity, the insignificant publication bias suggested that the results of included studies had depended mostly on the objective quality of the research.

Contradictory conclusions on validation of cell-free DNA assays for breast cancer screening have long existed, led by poor method standardization and variable analytical factors. Hence, the present study conducted comprehensive meta-analyses to evaluate the diagnostic accuracy of cfDNA assays. The results show high levels of accuracy of circulating DNA analyses, especially through qualitative assays. The overall accuracy of circulating DNA analysis was higher than the routinely used biomarkers CA15-3 and CA27.29 [68]. The mean sensitivity and specificity (0.88 and 0.98) of 6 studies applying more modern qualitative cfDNA assays were higher than those (0.87 and 0.89) of digital mammography, the current benchmark of breast cancer screening [4]. Although the likelihood ratio (LR) based on 24 studies showed imperfect robustness, the LR of a subgroup of 6 recent qualitative studies was satisfactory [39–41, 51–53]. Thus, these newly-emerging cfDNA tests are highly recommended as a complement to conventional cytological and histological examinations for breast cancer diagnosis.

Our meta-analysis had some limitations. First, it was impossible for us to determine all sources of heterogeneity. We did not include some covariates because the required data were not available from the selected articles. These probable covariates included tumor size, metastasis, TNM staging, flow, and timing of these studies. Second, though we performed a thorough literature search, a smaller number of studies were included in the qualitative analysis group, which might have weakened the statistical significance. Third, the inclusion of only English-language studies might have introduced bias to the analysis. Consequently, further longitudinal studies focusing on advanced molecular methods to characterize cell-free DNA in breast cancer are desired to support the results of our meta-analyses.

## MATERIALS AND METHODS

### Literature source and search

The studies included in this meta-analysis were independently retrieved and reviewed by two authors (Z Lin and J Neiswender). A systematic literature search was performed in PubMed, Web of Science, and Embase databases to identify eligible studies. Studies from different databases were imported to EndNote for further review. The search terms included “breast cancer”, “breast tumor”, “cell-free DNA”, “circulating DNA”, “plasma DNA”, “serum DNA”, “sensitivity and specificity”, and “accuracy”. No limit on start date for publications was applied, and only studies prior to 20 September 2016 were evaluated. Additional articles were identified by manually reviewing the references of included articles. When necessary, the authors of included articles were contacted for further study details.

### Inclusion and exclusion criteria

Studies that met the following criteria were included: (a) cohort studies that evaluated indicators originating from circulating cfDNA in plasma or serum; (b) sufficient data was presented for describing or calculating sensitivity and specificity values. Studies meeting any of the following criteria were excluded: (a) the article included specific evaluation indicators that were studied so rarely that they could not be included in a grouped analysis; (b) reviews, letters, technical reports, case reports, comments; (c) studies consisting of less than 10 breast cancer patients.

### Data extraction

Two reviewers (Z Lin and J Neiswender) independently extracted data from the included articles and integrated the final results with assistance from a third author (X Ma). Data extracted from the articles included lead author, publication year, participant characteristics, experimental methods, assay indicators, cutoff values, sensitivity and specificity data. True positive (TP), true negative (TN), false positive (FP), and false negative (FN) were also collected directly or calculated according to the sensitivity, specificity, positive predictive value (PPV) and negative predictive value (NPV) in every selected study.

### Statistical analysis

Standard methods for meta-analyses of diagnostic tests were performed [69] using statistical software programs (Stata, version 12.0; Stata Corporation; College Station; and Meta-Disc for Windows). To measure the accuracy of cfDNA assays, sensitivity and specificity, positive likelihood ratio (PLR), negative likelihood ratio (NLR), diagnostic odds ratio (DOR) were yielded by TP, FP, FN, TN from grouped studies. To summarize the overall accuracy, summary receiver operating characteristic (SROC) curves were constructed by the Moses-Shapiro-Littenberg method [70].

The term heterogeneity refers to the degree of variability in results across studies. Statistically significant heterogeneity among these studies was verified using likelihood ratio test (LRT)_I^2^ statistic [71] and LRT_Q (χ^2^) statistics. I^2^ ≥ 50% or *P* < 0.10 for LRT_Q indicates substantial heterogeneity. Meta-regression analysis was used to explore the sources of heterogeneity [72]. Covariates on DOR including sampling of patients and experimental methods were assessed. The relative DOR (RDOR) was calculated to analyze the change in diagnostic precision in the study per unit in the covariate [73]. The Deek's funnel plots were used to examine potential presence of publication bias [74]. For each analysis, a result was considered to be statistically significant if the *P*-value was < 0.05.

## CONCLUSIONS

In conclusion, our study is the first comprehensive meta-analysis on the overall accuracy of circulating cell-free DNA assays in breast cancer screening. This study suggested that the diagnostic accuracy of quantitative analysis of circulating DNA is better than conventional tumor biomarkers, and the accuracy of advanced qualitative analysis demonstrated even higher level of discriminatory power in breast cancer detection. Although the high specificity of qualitative methods is encouraging, further research must address ways to make this approach more sensitive through identification of more reliable cfDNA properties associated with breast cancer. Due to lack of robustness, the quantitative cfDNA assays cannot be used alone in cancer diagnosis without parallel cytological or histological examinations. Meanwhile, some modern qualitative assays of circulating cell-free DNA have strong potential to be applied as an independent tool to improve the benchmark for early breast cancer detection and diagnosis.

## SUPPLEMENTARY MATERIALS FIGURES AND TABLES



## Abbreviations

AUC: area under the curve
BPS: bisulphate pyrosequencing
CEA: carcinoembryonic antigen
cfDNA: cell-free DNA
CTC: circulating tumor cell
DOR: diagnostic odds ratio
ELISA: enzyme linked immunosorbent assay
FSGS: fluorochrome SYBR Gold stain
HCC: hepatocellular cancer
LRT: likelihood ratio test
LOH: loss of heterozygosity
NGS: next-generation sequencing
NLR: negative likelihood ratio
PLR: positive likelihood ratio
RT-qPCR: real-time quantitative PCR
RDOR: relative diagnostic odds ratio
SROC: summary receiver operating characteristic
TADS: tagged-amplicon deep sequencing
