# Supplementary material for: Value of circulating cell-free DNA analysis as a diagnostic tool for breast cancer: a meta-analysis
Source: Oncotarget. 2017 Feb 28;8(16):26625–36. doi: 10.18632/oncotarget.15775 (PMC5432284; doi:10.18632/oncotarget.15775)
Supplement: Supplementary file 1 [file oncotarget-08-26625-s001.pdf]

Value of circulating cell-free DNA analysis as a diagnostic tool for breast cancer: a meta-analysis

Supplementary Materials

Supplementary Table 1: Summary of sensitivity, specificity, PLR, NLR, DOR, AUC, LRT-I², LRT-Q, Spearman coefficient of study groups

	Studies	Sensitivity	Specificity	PLR	NLR
Breast cancer versus healthy controls					
Overall	24	0.70 (0.68–0.72)	0.87 (0.85–0.89)	6.22 (4.31–8.99)	0.25 (0.17–0.36)
Quantitative	14	0.78 (0.75–0.80)	0.83 (0.81–0.86)	4.83 (3.37–6.91)	0.22 (0.13–0.35)
Quant. Omit 3	11	0.79 (0.77–0.82)	0.83 (0.80–0.85)	5.07 (3.32–7.75)	0.17 (0.09–0.32)
Qualitative	10	0.50 (0.45–0.54)	0.98 (0.96–0.99)	16.52 (8.65–31.58)	0.32 (0.19–0.54)
Qual. Omit 4	6	0.88 (0.81–0.93)	0.98 (0.95–0.99)	24.46 (11.38–52.58)	0.14 (0.07–0.28)
Breast cancer versus benign disease					
Overall	8	0.75 (0.71–0.79)	0.79 (0.73–0.84)	2.40 (1.13–5.12)	0.29 (0.12–0.74)

	DOR	AUC	LRT-I²	LRT-Q	Spearman
Breast cancer versus healthy controls					
Overall	32.31 (17.35–60.18)	0.9314	78.60%	107.52 ($P < 0.001$)	–0.061 ($p = 0.777$)
Quantitative	24.40 (12.07–49.31)	0.9116	84.30%	82.76 ($P < 0.001$)	–0.191 ($p = 0.513$)
Quant. Omit 3	31.91 (13.65–74.62)	0.9193	87.40%	74.40 ($P < 0.001$)	–0.391 ($p = 0.235$)
Qualitative	68.45 (19.29–242.85)	0.9919	49.80%	17.94 ($P = 0.036$)	–0.383 ($p = 0.275$)
Qual. Omit 4	256.60 (77.40–850.69)	0.9886	0.00%	1.23 ($P = 0.942$)	0.029 ($p = 0.957$)
Breast cancer versus benign disease					
Overall	9.49 (1.76–51.03)	0.8213	91.20%	79.25 ($P < 0.001$)	–0.096 ($p = 0.821$)

PLR = positive likely ratio; NLR = negative likely ratio; DOR = diagnostic odds ratio; AUC = area under curve; LRT = likelihood ratio test; Spearman = Spearman correlation coefficient.
